# Intelligent Performance Evaluation in Rowing Sport Using a Graph-Matching Network

**DOI:** 10.3390/jimaging9090181

**Published:** 2023-08-31

**Authors:** Chien-Chang Chen, Cheng-Shian Lin, Yen-Ting Chen, Wen-Her Chen, Chien-Hua Chen, I-Cheng Chen

**Affiliations:** 1 Department of Computer Science and Information Engineering, Tamkang University, New Taipei City 25137, Taiwan; ccchen34@mail.tku.edu.tw (C.-C.C.); a80598059@gmail.com (Y.-T.C.); 2 Office of Physical Education, Tamkang University, New Taipei City 25137, Taiwan; 137540@mail.tku.edu.tw (W.-H.C.); 132098@mail.tku.edu.tw (C.-H.C.); masa@mail.tku.edu.tw (I.-C.C.); 3 Department of Physical Education and Sport Sciences, National Taiwan Normal University, Taipei City 11718, Taiwan

**Keywords:** OpenPose, graph neural network

## Abstract

Rowing competitions require consistent rowing strokes among crew members to achieve optimal performance. However, existing motion analysis techniques often rely on wearable sensors, leading to challenges in sporter inconvenience. The aim of our work is to use a graph-matching network to analyze the similarity in rowers’ rowing posture and further pair rowers to improve the performance of their rowing team. This study proposed a novel video-based performance analysis system to analyze paired rowers using a graph-matching network. The proposed system first detected human joint points, as acquired from the OpenPose system, and then the graph embedding model and graph-matching network model were applied to analyze similarities in rowing postures between paired rowers. When analyzing the postures of the paired rowers, the proposed system detected the same starting point of their rowing postures to achieve more accurate pairing results. Finally, variations in the similarities were displayed using the proposed time-period similarity processing. The experimental results show that the proposed time-period similarity processing of the 2D graph-embedding model (GEM) had the best pairing results.

## 1. Introduction

In recent years, many studies have been conducted that analyzed the performance of individual players in sports domains [1,2,3,4,5,6,7,8]. While there are many studies that focus on motion analysis, most require athletes to wear sensors to acquire their posture data; however, this method easily introduces noise due to friction during motions [1,2,8]. Therefore, the video-based contactless approach, which aims to acquire whole posture data using a simple camera without placing sensors on body parts, has made it easier and more convenient for scholars to analyze a variety of postures in many exciting sports domains [3,4,6,7].

Video-based motion analysis has witnessed numerous applications, including human–computer interaction systems [3], human action understanding systems [4], medical assistance systems [9], and human pose estimation [10], all based on deep learning and recognition models. The OpenPose system [10] has proven to be valuable in capturing the skeletal joints of individuals, even in complex postures, using a simple camera without the need for specialized hardware like the Kinect device [3,9]. However, as most previous applications are relevant only for recognizing simple postures, the issue of how to analyze the complex postures of sports players has been neither addressed nor effectively analyzed.

Several previous works [10,11,12,13,14,15,16,17,18,19,20,21,22] have utilized the OpenPose model [10] for various applications. Qiao et al. [11] used a series of coordinate trajectories of joint points to draw a curve that was used to determine whether Tai Chi movements were standard. Tsai et al. [12] estimated the depth distance between a person and a lens in a single image using the OpenPose model to capture the coordinates of the human body keypoints. Nakai et al. [13] proposed a prediction method for a basketball shooting system, which detected human body keypoints using the OpenPose model and predicted basketball free throw shooting using a logistic regression model. In addition, several applications based on deep learning have also been proposed. Toshev et al. [14] proposed a state-of-the-art approach based on deep neural networks (DNNs) for human pose estimation. Xiao et al. [15] proposed a simple baseline method based on ResNet architectures [16] as the backbone network for human pose estimation and tracking. Zhang et al. [17] proposed a golf analysis system that includes a human detection subsystem and a performance analysis subsystem. In the human detection subsystem, they applied the OpenPose model, human tracking, and an LSTM deep learning model to detect golf players’ postures, while the performance analysis subsystem scored the comparison results of the golf players’ postures. Theagarajan et al. [18] proposed an automatically generated visual analytics and player statistics system for soccer players based on convolutional neural networks (CNNs) and deep convolutional generative adversarial networks (DCGANs). These systems can successfully perform relevant applications using system models, such as predicting basketball shots, assessing the standardness of sports movements, etc. However, they still experience a decrease in accuracy when used in complex environments. For instance, object appearance variations and lighting changes, factors present in a complex environment, make it challenging for the systems to accurately capture and analyze information from images [19,20,21]. Moreover, the issue of pairing athletes to work together to enhance the performance of a sports team has not been addressed.

This study proposed a video-based performance analysis system for analyzing the performance of rowing pairs using a graph-matching network, which first detected and acquired human joint points using the OpenPose system from a rowing video. Then, the detected human joints were represented as graph structures that extracted the features of the rowing posture process. The rowing posture feature of each video frame was extracted using the graph-embedding model (GEM) and graph-matching network (GMN) model. Then, a video was developed to compare the baseline processes for two video sequences, and the performances of the rowing pairs were measured by calculating the rowing posture similarities in the pair using the GEM and GMN models. Finally, the proposed time-period similarity processing method was used to distinguish the degree of similarity changes in the players in the video segments. Experiments were carried out using a dataset of over 15 test rowing players, and the results show that the proposed approach can effectively evaluate the performances of rowing pairs and provide good suggestions for coaches when creating player groupings and training programs.

In the sport of rowing, consistency in the rowing movements among team members has a significant impact. Well-coordinated rowing motions contribute to achieving outstanding performance. Therefore, similarity in rowing postures among athletes has great significance. The purpose of this study is to analyze paired rowers using a graph-matching network to enhance the performance of a team sport. This study was organized into the following sections. Section 2 gives a brief review of related works, including the OpenPose system [10] and graph neural networks (GNNs) [18,19,20,21,22]. Section 3 provides the details of the proposed approach. Section 4 presents the experimental results, and Section 5 offers concluding remarks.

## 2. Related Works

This section briefly reviews some works related to this study, the OpenPose system [10] and graph neural networks (GNNs) [22,23,24,25,26], which are described in the following two subsections, respectively.

### 2.1. The OpenPose System

**Figure 1:**
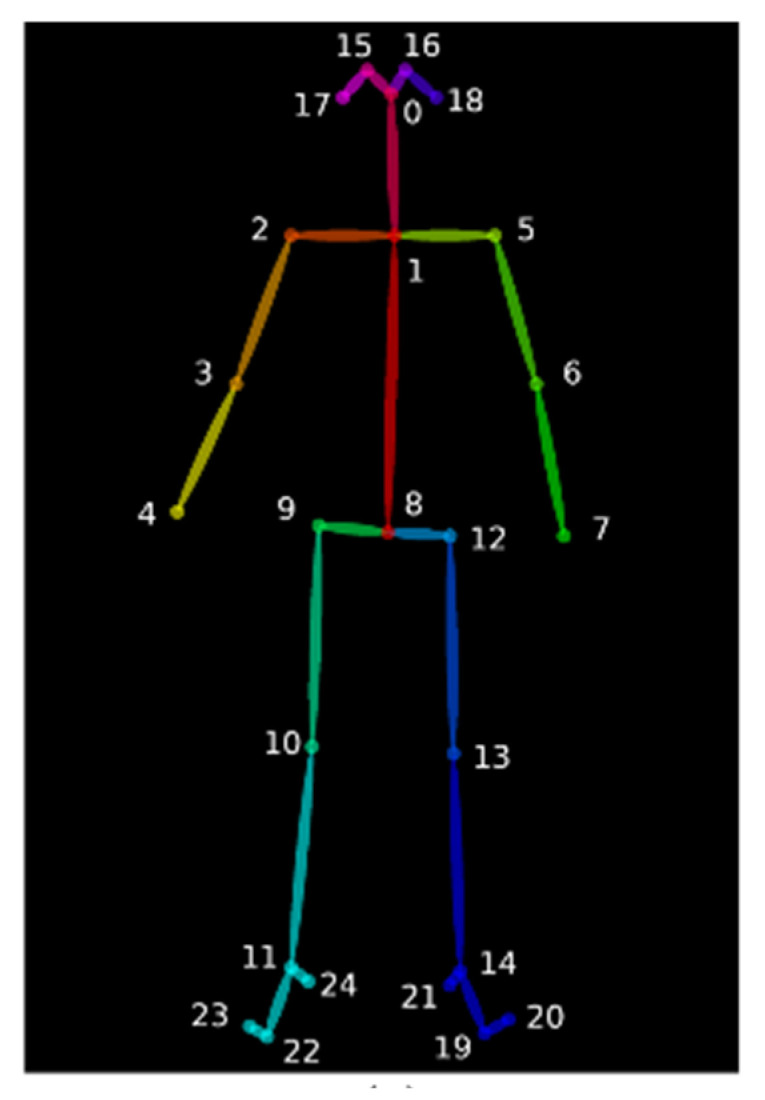
Keypoints detected using the OpenPose system and body25 model. Adapted from [10].

The OpenPose system, developed at Carnegie Mellon University, is a widely used, deep learning-based, real-time pose estimation system [10]. This system is capable of detecting multi-person poses in real-time by leveraging the COCO, MPII, and body25 models, with the number of human joints being determined based on the specific model used. Among these models, the OpenPose system with the body25 model yields more accurate keypoint results compared with the COCO and MPII models. Figure 1 illustrates detection results obtained using the OpenPose system with the body25 model. Each detected keypoint in the resulting image contains three pieces of information: the *X* coordinate, *Y* coordinate, and *C* confidence.

The OpenPose system is primarily built upon the human posture evaluation algorithm, with a core component known as part affinity fields (PAFs). This method uses a bottom-up detection approach, where it initially identifies joint point positions on the human body and subsequently extends to form the complete skeleton. As a result, the number of people being detected has minimal impact on the computational time required by OpenPose.

### 2.2. Graph Neural Networks

Deep learning has been extensively applied in image and sound recognition tasks, predominantly in Euclidean space recently. However, existing analysis methods like convolutional neural networks (CNNs) and recurrent neural networks (RNNs) encounter challenges when dealing with non-Euclidean spaces, such as matching human joint points. Consequently, researchers have introduced deep learning models based on graph neural networks (GNNs) [22,23,24,25,26] to tackle non-Euclidean spaces.

Although Sperduti and Starita initially proposed using neural networks for graph analysis in 1997 [20], it was not until 2005 that Gori et al. [25] presented a complete GNN architecture. Li et al. [26] further introduced the graph-embedding model (GEM) and graph-matching network (GMN), based on the GNN framework for graph-pairing tasks. GEM primarily embeds each graph into a low-dimensional vector using message transfer among adjacent nodes, allowing for distance calculation and similarity measurement between graphs. The graph-matching network (GMN), built upon the three-layer architecture of GEM, enhances accuracy in graph pairing by introducing a cross-graph message exchange mechanism alongside the node message propagation within the graph. Additionally, GNN has been extensively applied in various practical applications and research areas, including human movement recognition [27,28], identity analysis using motion [29], football prediction analysis [18,30], and similarity-based pairing tasks [31,32].

This study aimed to develop a video-based approach to analyze the performance of rowing poses of rowing pairs. To enable effective automatic comparison of rowing poses, the OpenPose system was used to extract robust rowing pose features and convert them into a graph structure that is robust to external factors, such as appearance (e.g., size, shape, and color) and lighting conditions. Subsequently, the GEM and GMN models were utilized to analyze the similarities in rowing postures between each pair of rowers.

## 3. The Proposed Approach

**Figure 2:**
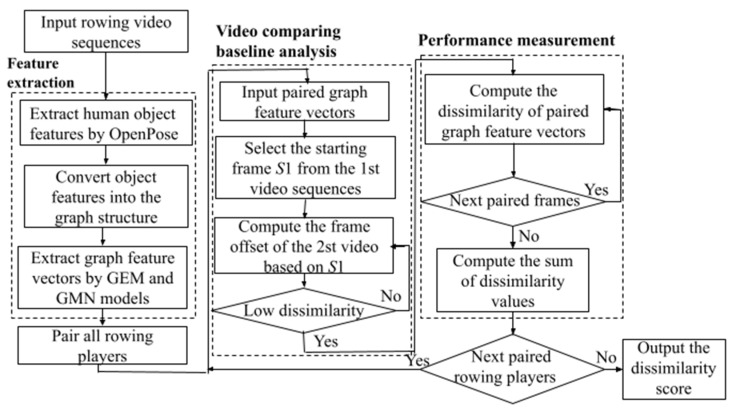
The proposed analysis approach for rowing player pairs.

This section presents details of the proposed approach for analyzing the performance of rowing pairs. As shown in Figure 2, the proposed approach consists of three main steps: feature extraction, baseline comparison analysis, and rowing performance measurement. The details of the proposed approach are described as follows.

### 3.1. Feature Extraction

This study used a video-based performance analysis system to analyze the performance of paired rowers, that is, the human body joints of the rowing pose features were detected and extracted using the OpenPose system. Furthermore, analyses were conducted to identify similarities in the rowing postures of paired rowers.

**Figure 3:**
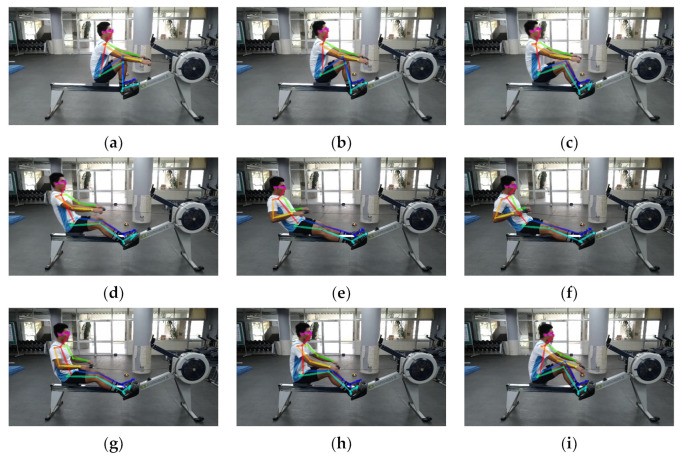
The human joint points detected during a rowing period using the OpenPose system. (**a**–**i**) The consecutive rowing poses from the catch position to the recovery position during a rowing period.

Let a video sequence contain *L* frames *f*_1_, …, *f_L_*, where each frame *f_i_*, *i*∈[1, *L*] contains an image of *M* × *N* pixels. In order to extract human object features of the rowing pose in each video frame, the proposed approach first utilizes the OpenPose system with the body25 model to detect the human joint points of rowing poses. The human joint points from the OpenPose system consist of three values, namely, *X* coordinates, *Y* coordinates, and confidence *C* (0.0 ≤ *C* ≤ 1.0). Therefore, the set of body joints in the *i*th frame is obtained ${\left\{{(X}_{i},Y_{i})\right\}}_{i=1}^{B}$, where *B* is the number of human body joints. Figure 3a–i shows the detected human joint points in the consecutive rowing poses from the catch position to the recovery position during a rowing period.

**Figure 4:**
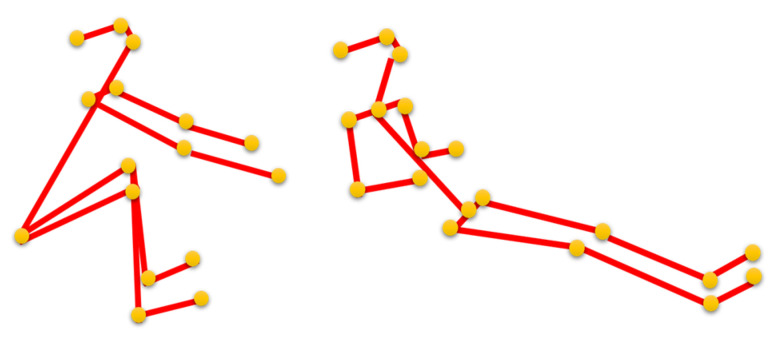
Illustration showing the detected rowing pose graph structures, which correspond to Figure 3a and Figure 3f, respectively.

After obtaining the rowing pose features of the human body joints, the features are presented as a graph structure in a graph-matching network (GMN) for further performance measurement. Figure 4 shows an example of two key rowing poses when rowing, the catch pose and the finish pose, which correspond to the detected graph structure of the rowing pose, as shown in Figure 3a and Figure 3f, respectively, where the joints of the human body are represented with vertices, and the connections in the joint points of the human body are represented with edges.

### 3.2. Baseline Comparison Analysis

To assess the performance of each pair of rowers, this study examined similarities between their rowing postures. The proposed system utilized a graph-matching network model, which contains a graph-embedding model (GEM) and a graph-matching network (GMN) [10], in order to extract the rowing posture feature vector from each video frame. Then, the similarities in the two rowing poses of each pair of video frames were calculated, which further demonstrated the performance of the paired rowers.

#### 3.2.1. The Input Data for the Model

In order to acquire the graph feature vectors (*GF*), twenty-five joint points were represented by graph structures, and for each rowing posture, the detected *X* and *Y* coordinates of human joints were input into the GEM and GMN models. Li et al. [26] proposed that the input space of GEM and GMN models required one-dimensional data, that is, the *X* and *Y* coordinates are respectively input into the graph-matching network. However, their study found that one-dimensional inputs were unable to distinguish between two rowing poses. Therefore, this research improved previous work by proposing a two-dimensional input space for the coordinates of human joint points.

Let the human joint point graph structures be *G_i_* (*V_i_*, *E_i_*), *i*∈[1, *L*], where *V_i_* are the *i*th human joint point nodes and *E_i_* is the set of edges that link the human joint point nodes. For the GEM and GMN models, the detected human joint points are formed as one-dimensional inputs ${\left\{X_{i}\right\}}_{i=1}^{L}$ and ${\left\{Y_{i}\right\}}_{i=1}^{L}$, respectively [26]. The proposed two-dimensional input data are $Input2D={\left\{{(X}_{i},Y_{i})\right\}}_{i=1}^{L}$. Therefore, the one-dimensional and two-dimensional human joint data were inputted into the GEM and GMN models to acquire each rowing pose feature for each frame, respectively.

#### 3.2.2. The Graph-Embedding Model (GEM)

The GEM and GMN models include a graph encoder layer, a propagation layer, and an aggregator layer [24]. In the graph encoder layer, feature encoding is performed on each node and edge of the graph, as follows:

(1) $$h_{i}=MLP\left(x_{Vi}\right),$$

(2) $$e_{ij}=MLP\left(x_{E_{ij}}\right),$$

where $x_{vi},\;\;x_{Eij},$ and MLP are the node feature vector, the edge feature vector, and the neural network of the hidden layer, respectively. It should be noted that if there is no available message for the edge and node, $x_{Vi}$ and $x_{Eij}$ are set to 1 [24].

To exchange node values in the propagation layer, each transfer generates a value, and each node $h_{i}^{t}$ receives values from adjacent nodes through the edges, resulting in the value of a new node $h_{i}^{t+1}$, which is defined as follows:

(3) $${Value}_{j->i}=f_{Value}\left(h_{i}^{t},h_{j}^{t},e_{ij}\right),$$

(4) $$h_{i}^{t+1}=f_{node}\left(h_{i}^{t},\sum_{j\in E}{Value}_{j\to i}\right),$$

where *f_value_* is a function based on *MLP* concatenation, *f_node_* is any core network of *MLP*, LSTM, or GRU, $h_{j}^{t}$ is the starting point of node value transfer, $h_{j}^{t}$ is the end point of receiving node values, and *e_ij_* is the edge that the node value passes through.

The aggregator layer aggregates nodes to obtain a graph feature vector *GF*, which is computed using:

(5) $$GF=MLP\left(\sum_{i\in V}\sigma \left({MLP}_{gate}\left(h_{i}^{T}\right)\right)⊙MLP\left(h_{i}^{T}\right)\right)$$

where ⊙ is the Hadamard product and *T* is the times of message passing. After the *T* round of value passing, the feature vectors of the rowing postures for each frame are obtained ${\left\{{GF}_{i}\right\}}_{i=1}^{L}$.

To calculate the dissimilarity in two rowing postures between each pair of frames for a pair of players, the proposed approach uses the Euclidean distance metric to calculate the dissimilarity score (*DISSIM_SCORE_*) between two rowing feature vectors *p*1 and *p*2, i.e.:

(6) $$DISSIM_{SCORE\left(p1,p2\right)}=\sum_{i=1}^{L}\frac{\sqrt{{\left({GF}_{i}^{p1}-{GF}_{i}^{p2}\right)}^{2}}}{L},$$

where *p*1 and *p*2 denote the number of paired rowers. In this study, 2 out of the 15 rowers were used for each similarity analysis. It was noted that lower value of the *DISSIM_SCORE_* denoted higher similarity between the rowing postures of two rowers, and it was more suitable for them to work together to improve the overall rowing performance, and vice versa.

#### 3.2.3. The Graph-Matching Network (GMN)

This study also extracted feature vectors from each video frame using a graph-matching network (GMN). The main difference between the GEM and GMN models is that the propagation layers in the GMN model exchange node messages in the graph and add a cross-graph message exchange mechanism. Let two graphs be $G_{1}=\left(V_{1},E_{1}\right)$ and $G_{2}=\left(V_{2},E_{2}\right)$. Each graph *G* = (*V*,*E*) represents a set of nodes *V* and edges *E*. Each node *i*∈*V* is associated with a feature vector *x_i_*, and each edge (*i*,*j*)∈*E* is associated with a feature vector *x_ij_*. In the propagation layer, the computation is defined as:

(7) $${Value}_{j->i}=f_{Value}\left(h_{i}^{t},h_{j}^{t},e_{ij}\right),\;\;for\;all\;(i,j)\in E_{1}\cup E_{2}$$

(8) $${Value}_{j->i}=f_{Value}\left(h_{i}^{t},h_{j}^{t},e_{ij}\right),\;\;for\;all\;(i,j)\in E_{1}\cup E_{2}$$

(9) $$h_{i}^{t+1}=f_{node}(h_{i}^{t},sum({Value}_{i}),sum(\mu_{i})),$$

where $sum({Value}_{i})$ represents the sum of all values from node *j* to node *i* and $sum(\mu_{i})$ represents the sum of all values from node *j*′ to node *i*, respectively. *f_match_* is a function of pairing cross-graph node value exchange, which is defined as,

(10) $$a_{j->i}=\frac{\exp(s_{h}(h_{i}^{t},h_{j}^{t}))}{\sum_{j^{\prime }}\exp(s_{h}(h_{i}^{t},h_{j^{\prime }}^{t}))},$$

where *s_h_*() is the Euclidean squared distance metric. In order to pair the most similar nodes in a graph with another graph, the difference value for all nodes should be computed, i.e.,

(11) $$\mu_{j->i}=a_{j->i}\times \left|h_{i}^{t}-h_{j}^{t}\right|,$$

and the total cross-graph value is:

(12) $$\sum_{j}\mu_{j->i}=h_{i}^{t}-\left(\sum_{j}a_{j->i}\right)\cdot h_{j}^{t},$$

where $a_{j->i}$ is the attention weight and $\sum_{j}\mu_{j->i}$ is the difference measured between $h_{i}^{t}$ and its closest neighbor in another graph.

Finally, the aggregator layer aggregates the nodes under each graph to obtain graph feature vectors *GF*1 and *GF*2, which are defined as follows:

(13) $$GF1=f_{G}({\left\{h_{i}^{T}\right\}}_{i\epsilon V_{1}}),$$

(14) $$GF2=f_{G}({\left\{h_{i}^{T}\right\}}_{i\epsilon V_{2}}).$$

Finally, the dissimilarity of the pair of rowing postures is computed using Equation (6).

It should be noted that, in order to accurately measure the similarity in each pair of rowing postures between two video sequences, the proposed system must measure the rowing poses in the same frame baseline; hence, this study proposed a video that compares baseline processing. First, the starting video frame *S*_1_ in the first video sequence *V*_1_ was selected; then, the corresponding lowest dissimilarity video frame was searched based on the starting video frame’s *S*_1_ of *V*_1_. The starting video frame of the second video *S*_2_ was obtained using:

(15) $$S_{2}=\underset{-100\leq \mathrm{fi}\leq 100}{\min}DISSIM\left(V_{1}\left({GF_{S}}_{1}\right),{\;V}_{2}\left({GF_{S}}_{1}+({GF_{S}}_{2}+f_{i})\right)\right),$$

where *GF* is the rowing feature vector, *f_i_* is the number of frame offsets, and *DISSIM*() is the dissimilarity function computed using the Euclidean distance.

### 3.3. Time-Period Similarity Processing

The purpose of the proposed time-period similarity processing (*TPS*) is to clearly distinguish the degree of similarity changes between the rowing postures of the paired rowers in the video segments.

In this study, the proposed approach performed segment similarity analyses in units of every 100 frames. The time-period similarity *TPS* was calculated as follows:

(16) $$TPS=\frac{\sum_{n=0}^{np-1}DISSIM(X)}{np}$$

where *np* is the average value of the segment of every 100 frames and *DISSIM*(*X*) is the dissimilarity measurement for each pair of rowing postures.

## 4. Results and Analysis

This section presents the experimental results of the proposed method. In this experiment, 15 high school rowers, who underwent training and practice in rowing techniques, used indoor rowing machines to simulate one minute of the actual rowing process [33,34], and each rower maintained a moderate rowing speed. Finally, we collected test videos from these 15 rowers to form the dataset used in our experiments. The test video was recorded at a speed of 60 frames per second (fps), and the resolution of each frame was 1920 × 1080. All experiments were run on a computer equipped with an Intel Core i9-10900k CPU, 16 G RAM, and NVIDIA GeForce RTX 2080 GPU and analyzed using the Python 3 software development tool.

### 4.1. The Rowing Posture Similarity Analysis

This study trained the graph neural networks 5000 times. Five rounds of node messages were exchanged each time, and the trained model was used to evaluate rowing posture similarities in the rowers. In the experiment, 15 rowers were numbered from 0 to 14, and after pairing and grouping, four models were used to calculate similarities in the rowing postures of the 2 rowers in each group. The models were as follows: 1D input of the graph-embedding model, 2D input of the graph-embedding model, 1D input of the graph-matching network, and 2D input of the graph-matching network.

Table 1 and Table 2 show the results of the rowing posture similarity analysis for all rower pairs using the 1D and 2D graph-embedding models (GEMs), respectively. In the dissimilarity matrix, which used the squared Euclidean distance defined in Equation (6) for similarity calculation, the red numbers indicate the lowest similarity in each column, while the green numbers indicate the highest similarity in each column. Furthermore, the lower the value, the greater the resemblance between the rowing postures. This indicates a higher suitability for assigning these rowers to the same group to further optimize their team’s performance in the sport.

### 4.2. Rowing Posture Analysis

**Figure 5:**
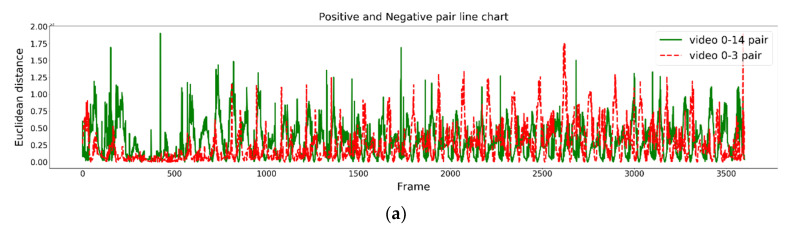

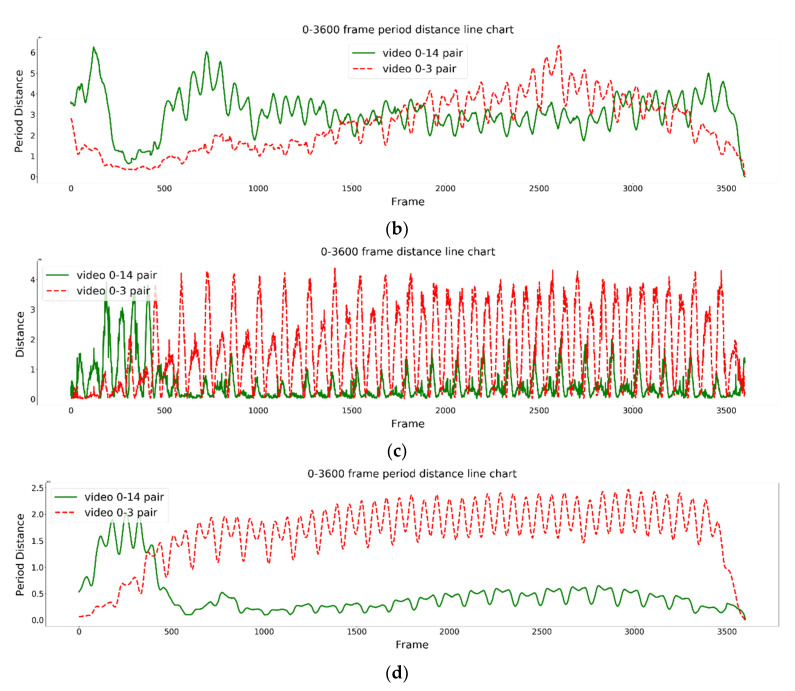
Experimental results of the 1D and 2D graph-embedding models (0–3600 frames): (**a**) 1D input GEM, (**b**) the time-period similarity results of (**a**), (**c**) 2D input of GEM, and (**d**) the time-period similarity results of (**c**).

**Figure 6:**
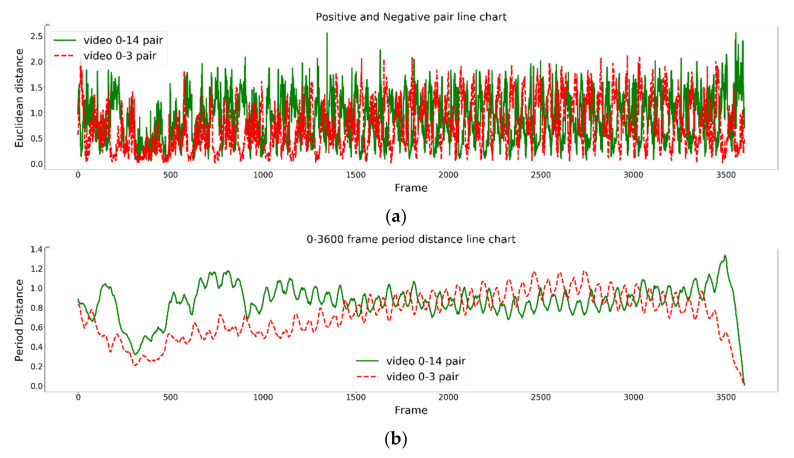

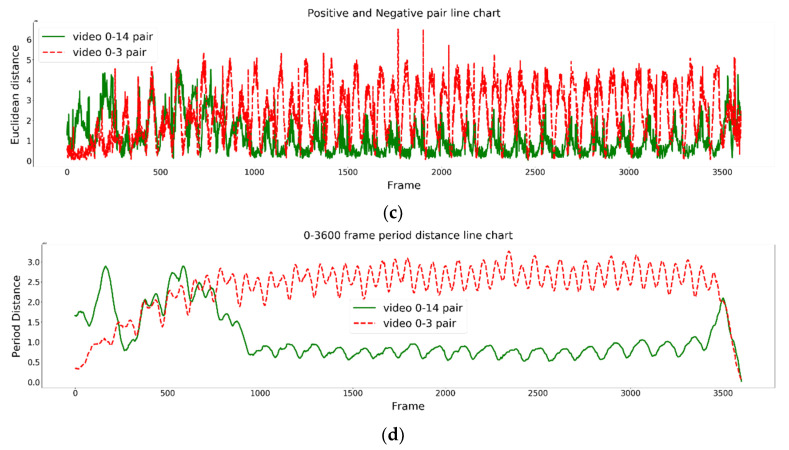
Experimental results of the 1D and 2D graph-matching networks (0–3600 frames): (**a**) 1D input GMN, (**b**) the time-period similarity results of (**a**), (**c**) 2D input GMN, and (**d**) the time-period similarity results of (**c**).

Figure 5 and Figure 6 show the posture similarity degree changes in the 1D and 2D inputs for the graph embedding models (GEMs) and graph-matching network (GMN), respectively, during the one-minute rowing process. The green solid line in the figure represents the most similar rower pair including No. 0 and No. 14, while the red dotted line represents the most dissimilar rower pair including No. 0 and No. 3. As shown in Figure 5a and Figure 6a, the differences in rowing postures between two rowers are not quite distinguished in the 1D input for GEM and GMN. However, the improved 2D input for GEM and GMN can slightly distinguish differences, as shown in Figure 5c and Figure 6c. Furthermore, our proposed time-period similarity analysis approach can significantly distinguish temporal differences in rowing motions, as shown in Figure 5b,d and Figure 6b,d. Moreover, Figure 5c,d and Figure 6c,d show that in the initial 500 frames, the posture similarities between rowers No. 0 and No. 3, which had the worst similarity originally, were relatively similar during this period. However, their differences in posture similarities became larger after about 500 frames. In contrast, rowers No. 0 and No. 14 maintained high similarities after 500 frames.

**Figure 7:**
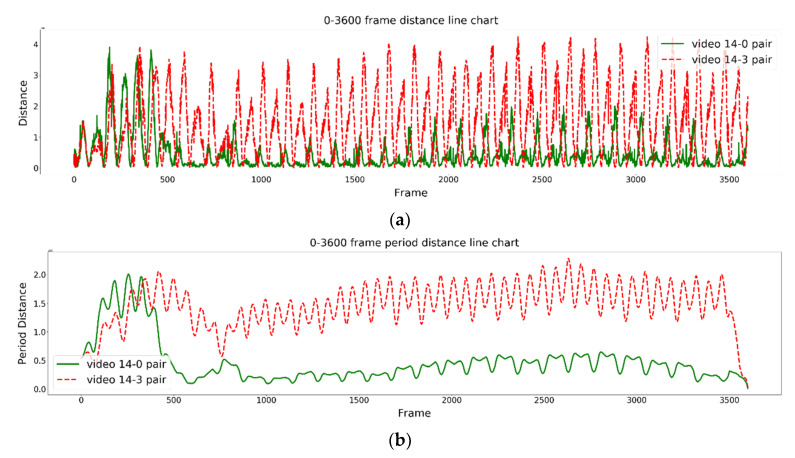
Experimental results for rower numbers 14, 0, and 3. (0–3600 frames): (**a**) 2D input GEM, which (**b**) is the proposed time-period similarity results of (**a**).

Figure 7 shows the similarities in the rowing posture results for two pairs of rowers regarding the 2D input for the graph-embedding model (GEM) at frame numbers 0~3600. The green solid line in the figure represents the rowing pair with the most similar rowing postures, while the red dotted line represents the rowing pair with the most dissimilar rowing postures. Figure 7a shows the rowing posture similarities in paired rowers using the 2D input GEM, while Figure 7b shows the proposed time-period similarity analysis results of Figure 7a. It can be found that the rowing postures of the two rowers were not stable before frame number 500 at the beginning of rowing; however, in the middle part of the video, the rowing postures of the two rowers became stable, as marked by the green line.

### 4.3. Visual Validation

In order to verify the effectiveness of the approach proposed in this study, skeletons of the rowing postures for the two groups of rowers were superimposed, their posture similarities were visualized for comparison, and 0~3600 frames were used for each group. Group 1 consisted of rowers No. 0 and No. 3 and had the lowest rowing posture similarities, while Group 2 consisted of rowers No. 0 and No. 14 and had the highest rowing posture similarities.

**Figure 8:**
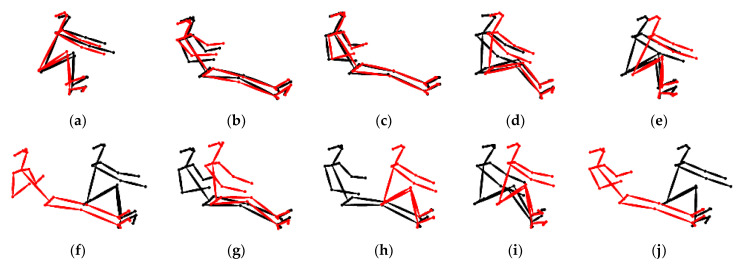
Rower numbers 0 and 3: (**a**–**e**) results comparing every 30 frames after frame number 0 and (**f**–**j**) results comparing every 30 frames after frame number 500.

Figure 8 shows the results of superimposing the skeletons of rowers No. 0 and No. 3 with the lowest rowing posture similarities, in which the red is rower No. 0 and the black is rower No. 3. Figure 8a–e shows that at the beginning of rowing, i.e., frame numbers 0~500, the rowing postures of the skeletons were matched because the rowing rhythms of the two rowers were almost the same. It should be noted that the initial motions in the rowing posts of rower No. 0 and rower No. 3 and their rhythms are very similar. However, as time progressed and physical exertion increased, their rowing poses and rhythms could not be consistently maintained, resulting in a gradual dissimilarity in their rowing poses over time. This observation is in line with the prediction results of the rowing posture analysis using the 2D GMN analysis model in the previous section [26]. In addition, Figure 8f–j shows that the rowing postures of the two rowers were already quite different; hence, the skeletons in the rowing postures did not match after frame number 500.

**Figure 9:**
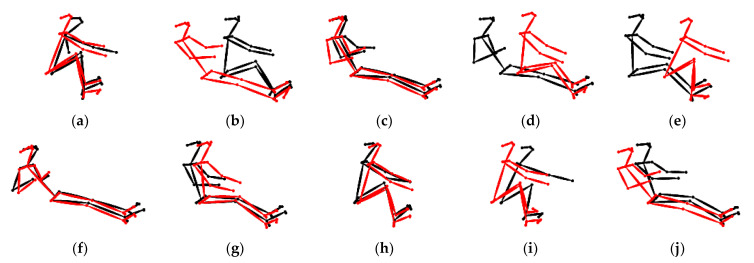
Rower numbers 0 and 14. (**a**–**e**) Results comparing every 30 frames after frame number 0 and (**f**–**j**) results comparing every 30 frames after frame number 500.

Figure 9 shows the results of superimposing the skeletons for rower No. 0 and No. 14, which had the highest rowing posture similarities, where red is rower No. 0 and black is rower No. 14. Figure 9a–e shows that the rowing postures of the two rowers were not well matched at the beginning of rowing, i.e., frame numbers 0~500. However, the skeletons of their rowing postures began to match after frame number 500, as shown in Figure 9f–j. It should be noted that the initial motion of rower No. 0’s rowing pose was standard, but their rowing rhythm could not be consistently maintained. On the other hand, rower No. 14’s rowing pose at the beginning of rowing was not quite standard, but their rowing rhythm was stable. Due to this, rowers No. 0 and No. 14 were not well-matched at the beginning of rowing. However, as the rowing rhythm was adjusted, the rowing poses of the two individuals gradually became more similar. The results of this experiment show that the similarity values obtained using this research method were all in line with the results of the visualized superimposed skeleton comparison. The final similarity matrix can be used to judge whether any two rowers are suitable to be grouped to increase the rowing speed.

## 5. Conclusions

This study presented an efficient approach for evaluating the performance of rowing pairs using a graph-matching network. The proposed approach used the OpenPose system to obtain the rowers’ postures during the rowing process and acquire the positions of human joints. Afterward, the 2D coordinates of the detected human joints were input into GEM and GMN models to extract the feature vectors of each rowing posture. The proposed video baseline analysis results calculate the performance of the rowing pairs. Furthermore, the similarities in each pair of rowing postures were efficiently calculated using the GEM and GMN models. The proposed time-period similarity analysis clearly distinguished the degree of similarity changes in rowers over time. This experiment was carried out to demonstrate that the proposed approach could effectively evaluate the performance of rowing pairs and provide good suggestions for coaches when creating rower grouping and training.

The main limitation of the proposed approach is that this study only simulates indoor rowing using indoor rowing machines, which may impose several limitations when applied to actual outdoor rowing scenarios. The primary strength of this study lies in the novel approach using graph-embedding models and graph-matching networks for rowing posture analysis. Despite its focus on rowing, this system can also be applied to other sports, thereby enhancing overall team performance. In the future, identifying how to use 3D posture features and integrating a 3D graph neural network model to improve the proposed system merit further study.

## Figures and Tables

**Table 1:** The dissimilarity matrix among all player pairs using 1D input graph-embedding model $\left(\times 10^{7}\right)$. The green numbers indicate the most similar pairing results, while the red numbers represent the least similar pairing results.

No.	0	1	2	3	4	5	6	7	8	9	10	11	12	13	14
**0**		2.93	2.40	4.89	2.11	2.83	2.32	4.15	3.26	2.84	3.85	4.03	2.83	3.29	1.43
**1**	2.93		2.28	3.21	2.65	2.79	2.39	3.17	2.90	3.01	3.36	3.11	3.50	3.19	2.63
**2**	2.40	2.28		3.39	2.39	2.87	2.68	3.37	3.05	3.13	3.51	2.72	3.21	3.05	2.38
**3**	4.89	3.21	3.39		3.09	3.25	2.34	2.43	3.33	3.67	2.54	1.23	5.14	3.36	3.92
**4**	2.11	2.65	2.39	3.09		2.77	1.40	4.85	4.32	2.66	4.45	2.86	2.25	3.38	2.71
**5**	2.83	2.79	2.87	3.25	2.77		2.55	2.56	2.44	2.31	3.73	3.20	2.87	3.16	2.47
**6**	2.32	2.39	2.68	3.25	1.40	2.55		3.09	2.95	1.53	2.90	2.75	3.02	2.64	1.85
**7**	4.15	3.17	3.37	2.43	4.85	2.56	3.09		2.08	2.70	1.90	2.32	5.06	2.62	2.48
**8**	3.26	2.90	3.05	3.33	4.32	2.44	2.95	2.08		2.68	2.61	3.77	3.41	2.92	2.22
**9**	2.84	3.01	3.13	3.67	2.66	2.31	1.53	2.70	2.68		3.66	3.61	3.35	2.64	2.17
**10**	3.85	3.36	3.51	2.54	4.45	3.73	2.90	1.90	2.61	3.66		2.50	6.98	3.84	2.55
**11**	4.03	3.11	2.72	1.23	2.86	3.20	2.75	2.32	3.77	3.61	2.50		5.04	3.12	3.62
**12**	2.83	3.50	3.21	5.14	2.25	2.87	3.02	5.06	3.41	3.35	6.98	5.04		3.46	3.15
**13**	3.29	3.19	3.05	3.36	3.38	3.16	2.64	2.62	2.92	2.64	3.84	3.12	3.46		2.51
**14**	1.43	2.63	2.38	3.92	2.71	2.47	1.85	2.48	2.22	2.17	2.55	3.62	3.15	2.51	

**Table 2:** The dissimilarity matrix among all player pairs using 2D input graph-embedding model $\left(\times 10^{7}\right)$. The green numbers indicate the most similar pairing results, while the red numbers represent the least similar pairing results.

No.	0	1	2	3	4	5	6	7	8	9	10	11	12	13	14
**0**		10.14	8.01	16.40	6.97	10.22	8.24	13.67	11.70	10.31	11.33	15.09	8.86	11.63	4.77
**1**	10.14		7.95	10.89	9.13	10.47	8.44	10.93	10.59	11.43	10.58	12.19	11.48	11.70	9.71
**2**	8.01	7.95		11.03	7.62	10.65	9.31	11.32	10.85	11.71	10.70	10.46	9.91	10.90	8.15
**3**	16.40	10.89	11.03		9.96	11.48	7.97	8.01	11.62	13.20	7.76	3.37	17.37	11.48	14.75
**4**	6.97	9.13	7.62	9.96		9.59	4.34	15.88	14.43	9.09	13.21	10.38	7.01	11.18	9.50
**5**	10.22	10.47	10.65	11.48	9.59		9.64	9.31	9.49	9.32	12.34	13.29	9.37	123.1	9.52
**6**	8.24	8.44	9.31	7.97	4.34	9.64		11.13	11.05	4.65	8.35	10.54	11.08	9.49	7.38
**7**	13.67	10.93	11.32	8.01	15.88	9.31	11.13		7.04	9.76	5.73	8.39	16.02	9.29	8.60
**8**	11.70	10.59	10.85	11.62	14.43	9.49	11.05	7.04		10.38	8.15	15.20	10.99	10.74	8.13
**9**	10.31	11.43	11.71	13.20	9.09	9.32	4.65	9.76	10.38		12.15	15.28	11.26	10.22	8.00
**10**	11.33	10.58	10.70	7.76	13.21	12.34	8.35	5.73	8.15	12.15		8.22	20.63	12.44	7.56
**11**	15.09	12.19	10.46	3.37	10.38	13.29	10.54	8.39	15.20	15.28	8.22		18.73	12.36	14.59
**12**	8.86	11.48	9.91	17.37	7.01	9.37	11.08	16.02	10.99	11.26	20.63	18.73		11.22	11.05
**13**	11.63	11.70	10.90	11.48	11.18	123.1	9.49	9.29	10.74	10.22	12.44	12.36	11.22		9.02
**14**	4.77	9.71	8.15	14.75	9.50	9.52	7.38	8.60	8.13	8.00	7.56	14.59	11.05	9.02	

## Data Availability

Not applicable.
